# Supplementary material for: Association of circulating branched-chain amino acids with risk of pre-diabetes: a systematic review and meta-analysis
Source: PeerJ. 2025 Sep 25;13:e20054. doi: 10.7717/peerj.20054 (PMC12476861; doi:10.7717/peerj.20054)
Supplement: Supplemental Information 2 — Note: Each study was assessed from three perspectives, including the selection of study groups, which provided a score of between 0 and 4 points; comparability of groups (0–2 points); and ascertainment of outcome (0–3 points). Studies with more than six points were considered high quality. Studies with a score of more than 6 are considered to be of high quality. Studies with scores of 4-6 are of moderate quality. Studies with a score below 4 are of low quality. [file peerj-13-20054-s002.docx]

**Table S1 The quality assessments of each included study**

| Study | Selection | | | | Comparability | Exposure | | | Score |
| --- | --- | --- | --- | --- | --- | --- | --- | --- | --- |
|  | Adequate definition of cases | Representativeness of the cases | Selection of controls | Definition of controls |  | Ascertainment of exposure | Same method of ascertainment for cases and controls | Non-Response rate |  |
| Xu-2013 | ★ | ★ | ★ | ★ | ★☆ | ★ | ★ | ★ | 8 |
| Owei-2019 | ★ | ★ | ★ | ★ | ★☆ | ★ | ★ | ★ | 8 |
| Li-2021 | ★ | ★ | ★ | ☆ | ★☆ | ☆ | ★ | ★ | 6 |
| Lee-2018 | ★ | ★ | ★ | ★ | ★☆ | ★ | ★ | ★ | 8 |
| Hu-2022 | ★ | ★ | ☆ | ☆ | ★☆ | ★ | ★ | ★ | 6 |
| Connelly-2017 | ★ | ★ | ★ | ★ | ★☆ | ★ | ★ | ★ | 8 |
| Andersson-Hall-2018 | ★ | ☆ | ★ | ☆ | ★☆ | ★ | ★ | ★ | 6 |
| Shi-2021 | ★ | ★ | ★ | ★ | ★☆ | ★ | ★ | ★ | 8 |
| Mook-2016 | ★ | ★ | ☆ | ☆ | ★☆ | ★ | ★ | ★ | 6 |
| Menge-2010 | ★ | ★ | ☆ | ★ | ★★ | ★ | ★ | ★ | 8 |
| Kujala-2016 | ★ | ☆ | ★ | ★ | ★☆ | ★ | ★ | ★ | 7 |
| Gaike-2020 | ★ | ☆ | ★ | ☆ | ★☆ | ☆ | ★ | ★ | 5 |
| Tulipani-2016 | ★ | ★ | ★ | ★ | ★☆ | ★ | ★ | ☆ | 7 |
| Safari-Alighiarloo-2024 | ★ | ★ | ★ | ☆ | ★☆ | ★ | ★ | ★ | 7 |
| Cobb-2015 | ★ | ★ | ★ | ★ | ★☆ | ★ | ★ | ★ | 8 |
| Mels-2013 | ☆ | ★ | ☆ | ☆ | ★☆ | ★ | ★ | ★ | 5 |

Note: Each study was assessed from three perspectives, including the selection of study groups, which provided a score of between 0 and 4 points; comparability of groups (0–2 points); and ascertainment of outcome (0–3 points). Studies with more than six points were considered high quality. Studies with a score of more than 6 are considered to be of high quality. Studies with a score of 4-6 are of moderate quality. Studies with a score below 4 are of low quality.
