# Supplementary figures and images for: Association of circulating branched-chain amino acids with risk of pre-diabetes: a systematic review and meta-analysis
Source: PeerJ. 2025 Sep 25;13:e20054. doi: 10.7717/peerj.20054 (PMC12476861; doi:10.7717/peerj.20054)

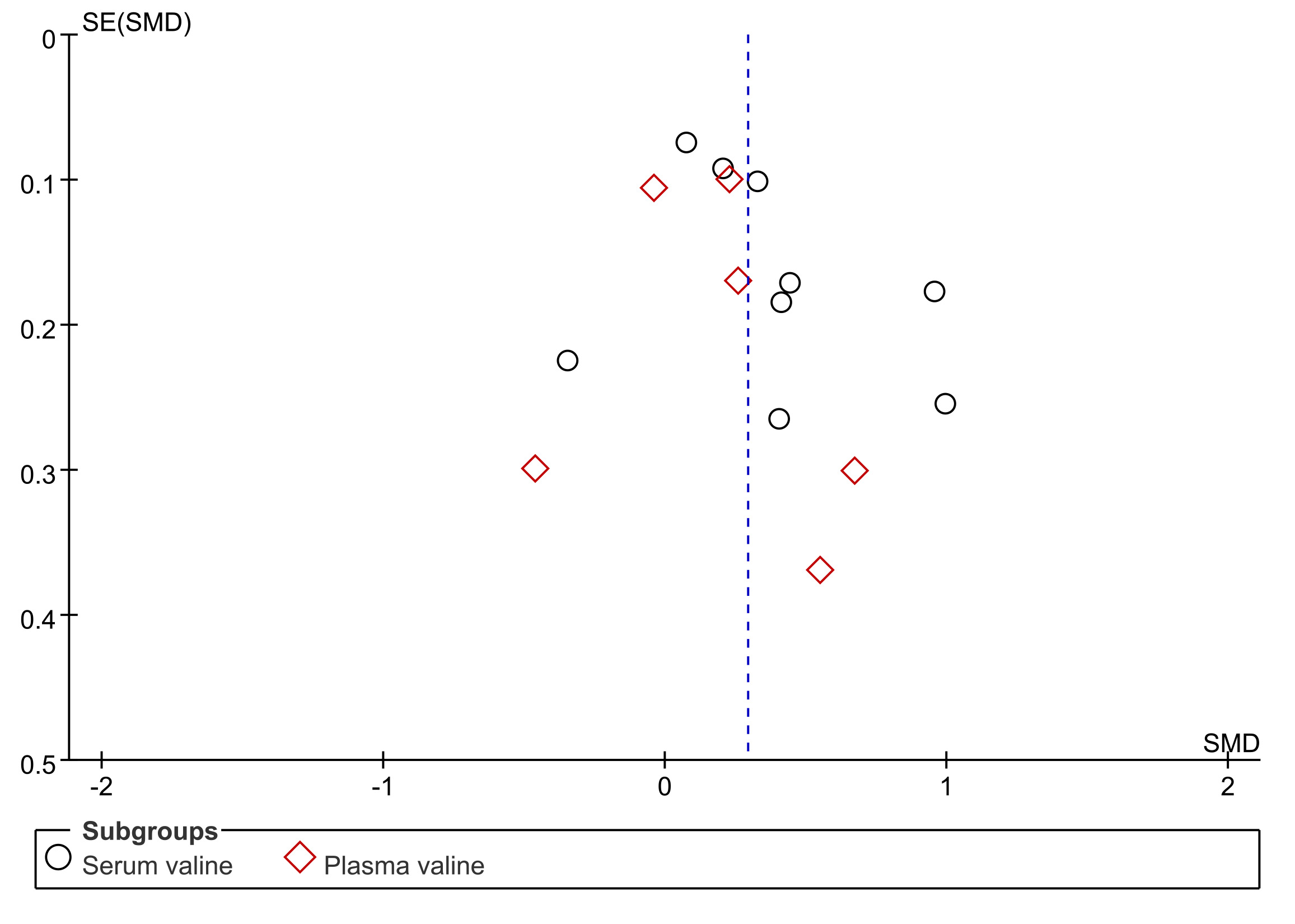

Supplement: Supplemental Information 3 [file peerj-13-20054-s003.jpg]

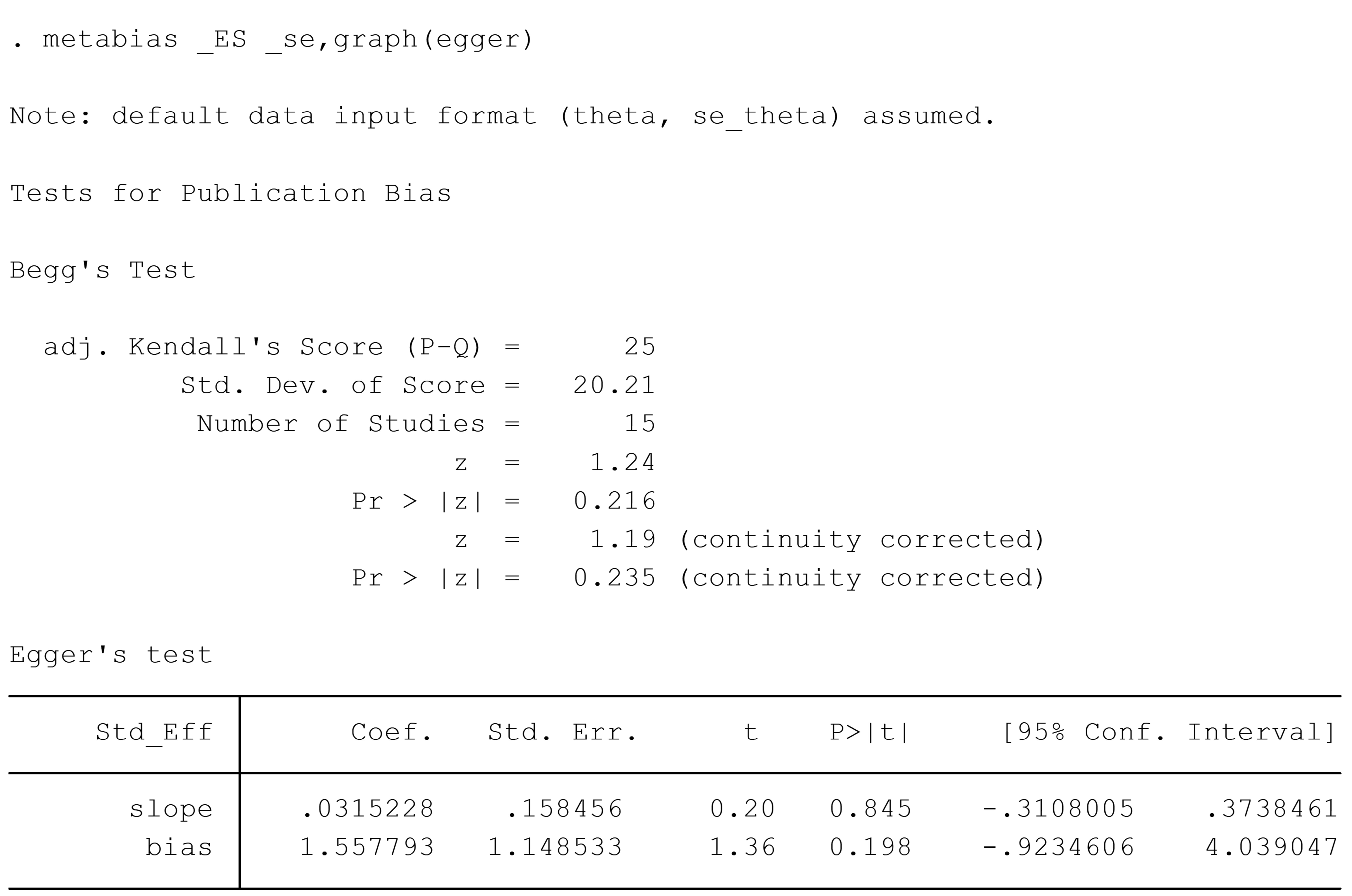

Supplement: Supplemental Information 4 [file peerj-13-20054-s004.jpg]

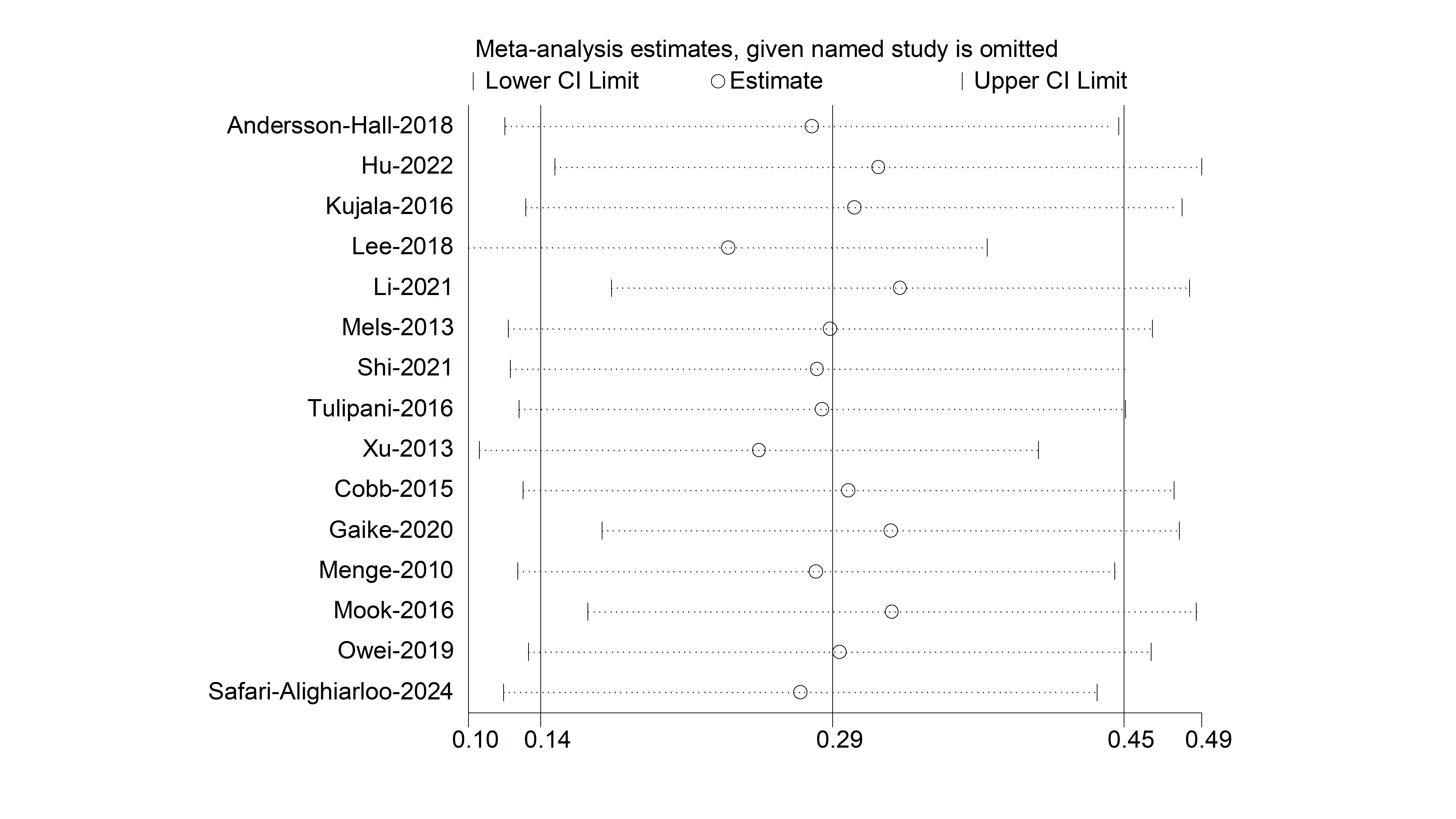

Supplement: Supplemental Information 5 [file peerj-13-20054-s005.jpg]

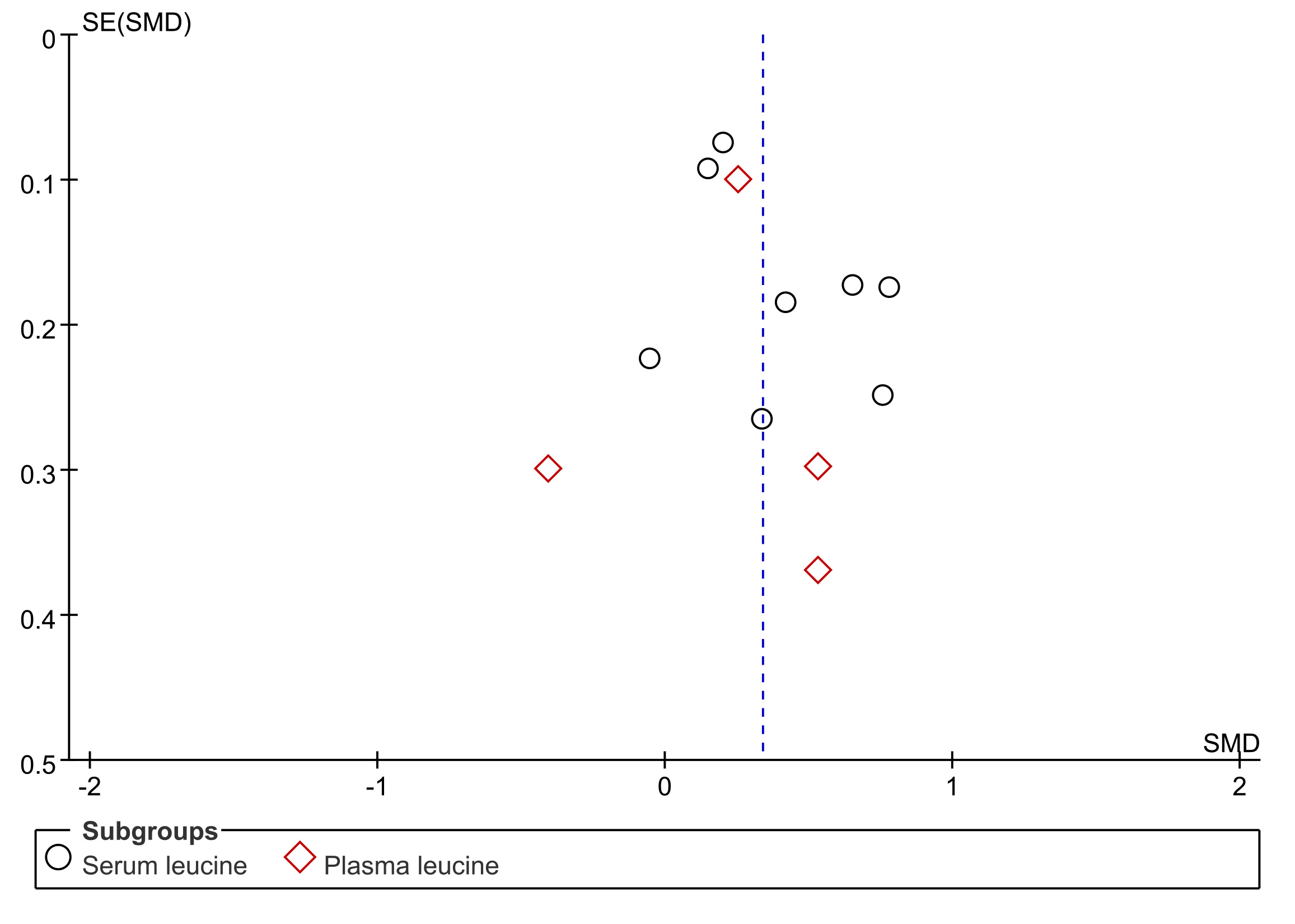

Supplement: Supplemental Information 6 [file peerj-13-20054-s006.jpg]

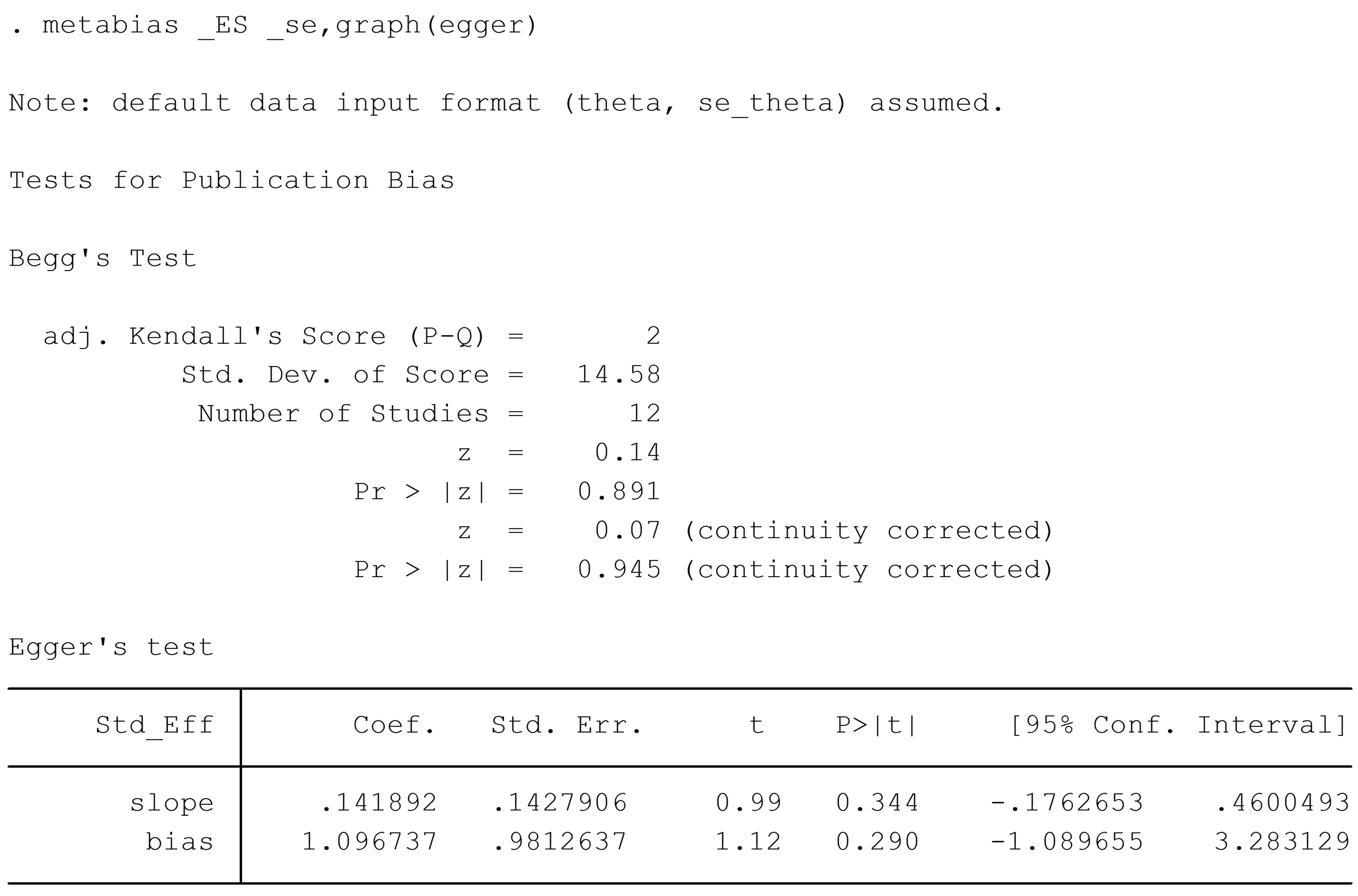

Supplement: Supplemental Information 7 [file peerj-13-20054-s007.jpg]

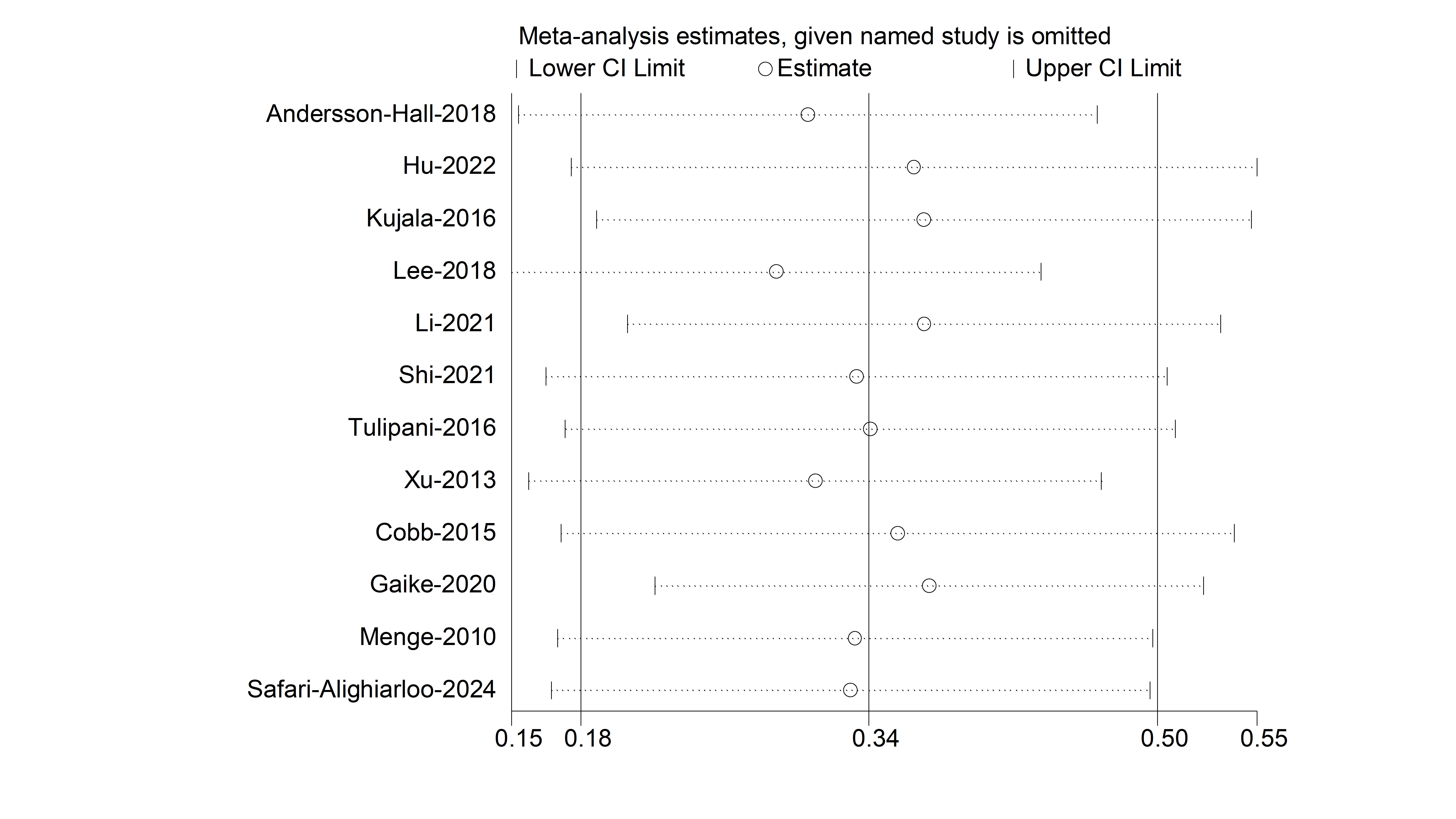

Supplement: Supplemental Information 8 [file peerj-13-20054-s008.jpg]

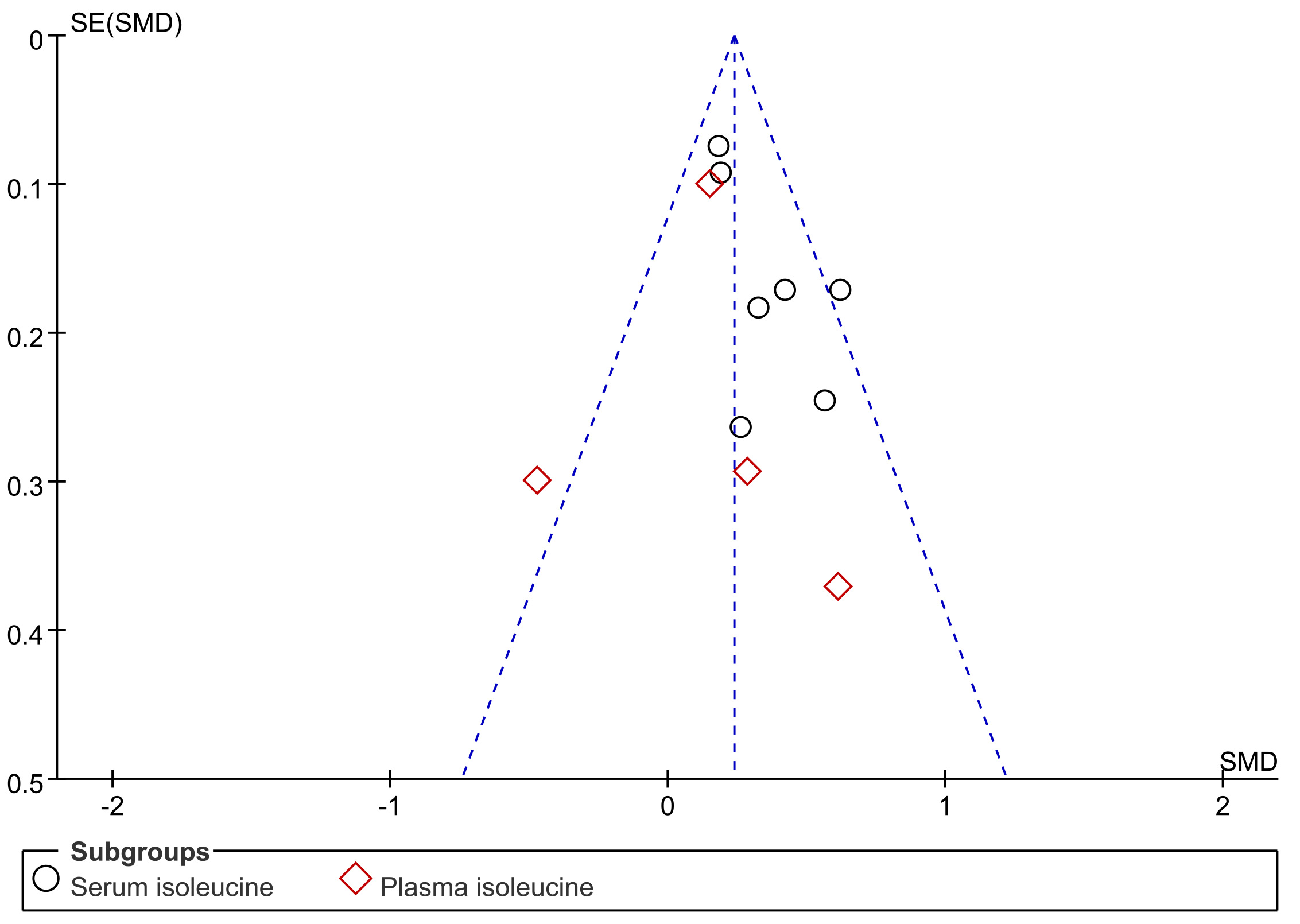

Supplement: Supplemental Information 9 [file peerj-13-20054-s009.jpg]

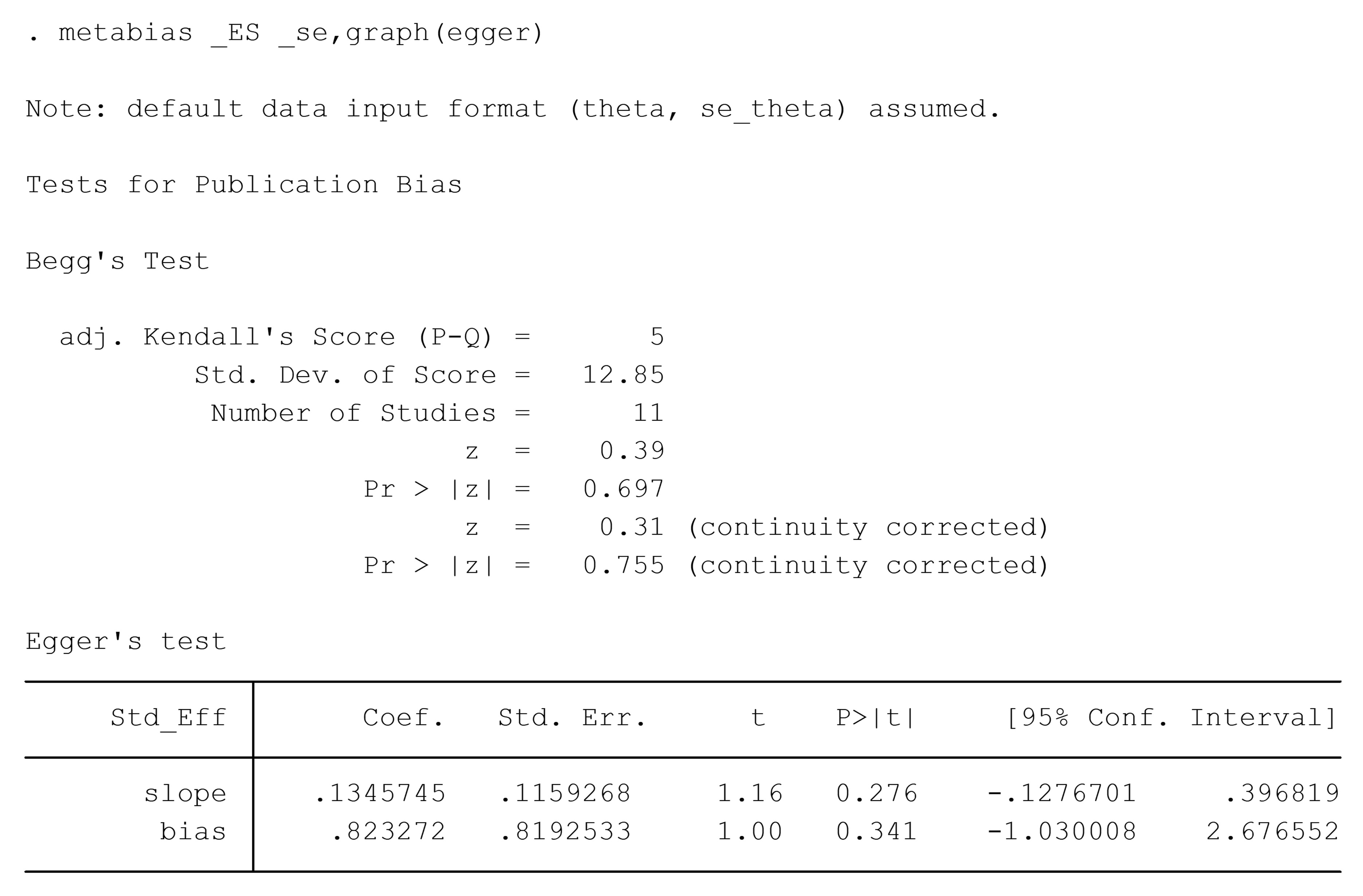

Supplement: Supplemental Information 10 [file peerj-13-20054-s010.jpg]

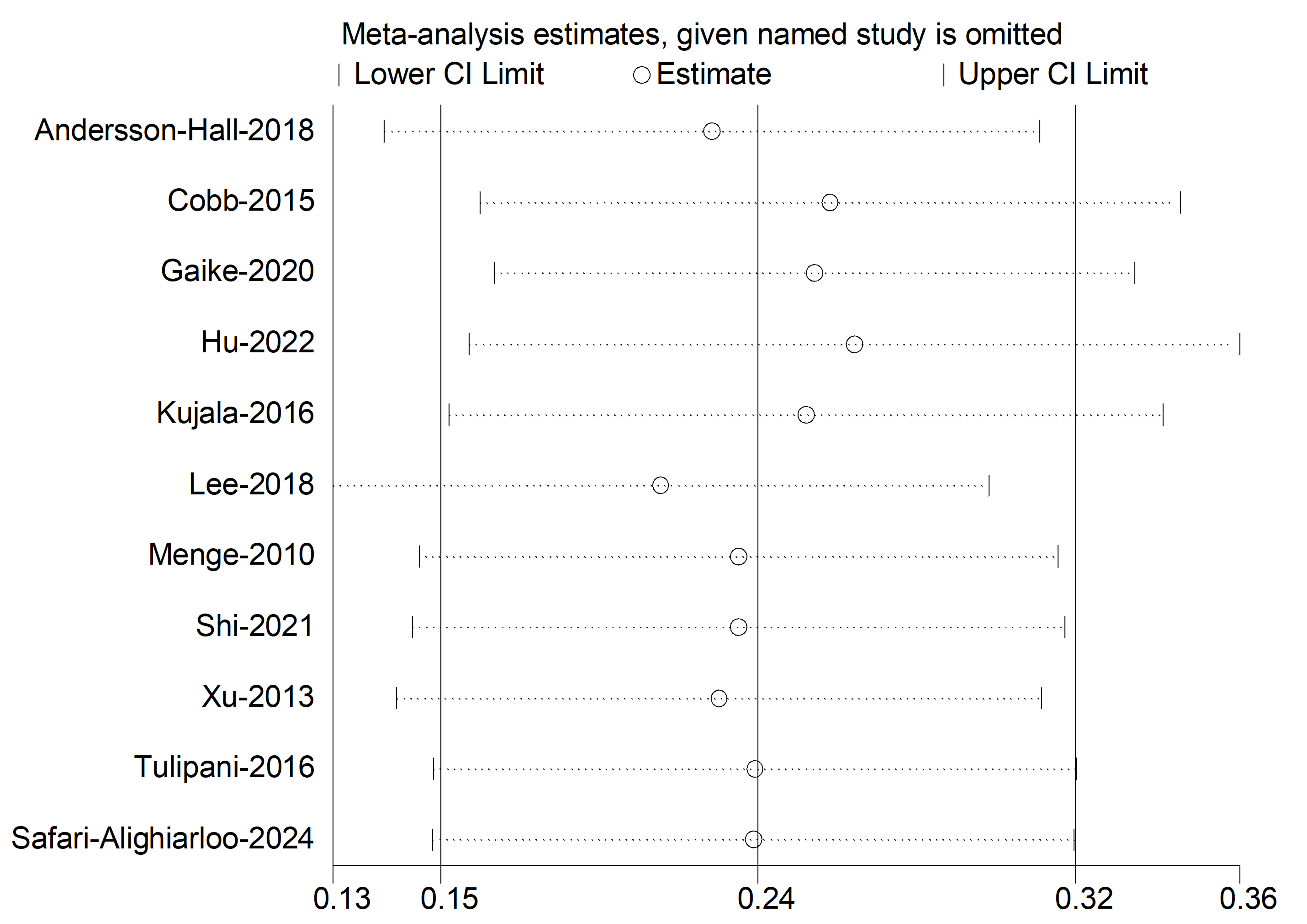

Supplement: Supplemental Information 11 [file peerj-13-20054-s011.jpg]
