# Supplementary material for: Association of circulating branched-chain amino acids with risk of pre-diabetes: a systematic review and meta-analysis
Source: PeerJ. 2025 Sep 25;13:e20054. doi: 10.7717/peerj.20054 (PMC12476861; doi:10.7717/peerj.20054)
Supplement: Supplemental Information 12 [file peerj-13-20054-s012.docx]

Supplementary files

# Supplementary Material 1

**Supplementary Material 1 The rationale for conducting the meta-analysis**

A growing body of research has explored the connection between circulating branched-chain amino acid levels and PreDM events, while their findings have been subject to considerable debate. This study is the first to systematically investigate the association between circulating branched-chain amino acid levels and pre-diabetes in a large sample size through a meta-analysis. It aims to address existing controversies in the field.

# Supplementary Material 2

**Supplementary Material 2 The contribution that the meta-analysis makes to knowledge in light of previously published related reports, including other meta-analyses and systematic reviews.**

Previous systematic reviews and meta-analyses have examined the relationship between metabolites and diabetes or pre-diabetes. However, these studies did not specifically investigate the association between pre-diabetes and branched-chain amino acid levels and were constrained by small sample sizes. This study is the first to systematically review and meta-analyze the association between prediabetes and BCAA levels using a large sample size. The study was characterized by a comprehensive search and strict inclusion and exclusion criteria. In addition, the study included subjects from multiple countries and regions, which better reflects the status of pre-diabetes in humans. This work is expected to have a significant impact on the field and clinical practice related to diabetes. It is emphasized that this manuscript is original and has not been published elsewhere. This study aims to provide valuable insights for clinicians managing prediabetes and for researchers investigating metabolic biomarkers for early detection. By synthesizing evidence comprehensively, it offers guidance for academic research, practical healthcare strategies, and policymaking.

# Supplementary Material 3

**Supplementary Material 3 Database search strategy**

**PubMed**

(("Prediabetic State"[Mesh]) OR (((((((((((Prediabetic States[Title/Abstract]) ) OR (State, Prediabetic[Title/Abstract])) OR (States, Prediabetic[Title/Abstract])) OR (Prediabetes[Title/Abstract])) OR (prediabetic[Title/Abstract])) OR (insulin resistance[Title/Abstract])) OR (HOMA-IR[Title/Abstract])) OR (Impaired glucose tolerance[Title/Abstract])) OR (glucose intolerance[Title/Abstract])) OR (pre-diabetes[Title/Abstract]))) AND (("Amino Acids, Branched-Chain"[Mesh]) OR (((((((((((Acids, Branched-Chain Amino[Title/Abstract]) OR (Branched-Chain Amino Acids[Title/Abstract])) OR (Amino Acids, Branched Chain[Title/Abstract])) OR (Branched-Chain Amino Acid[Title/Abstract])) OR (Acid, Branched-Chain Amino[Title/Abstract])) OR (Amino Acid, Branched-Chain[Title/Abstract])) OR (Branched Chain Amino Acid[Title/Abstract])) OR (BCAA[Title/Abstract])) OR (Isoleucine[Title/Abstract])) OR (leucine[Title/Abstract])) OR (Valine[Title/Abstract]))) Filters: Humans

**Embase**

#1. 'impaired glucose tolerance'/exp OR 'impaired glucose tolerance' OR 'prediabetic states':ab,ti OR 'state, prediabetic':ab,ti OR 'states, prediabetic':ab,ti OR 'prediabetes':ab,ti OR 'prediabetic':ab,ti OR 'insulin resistance':ab,ti OR 'homa-ir':ab,ti OR 'impaired glucose tolerance':ab,ti OR 'glucose tolerance':ab,ti OR

#2. 'branched chain amino acid'/exp OR 'acids,branched-chain amino':ab,ti OR 'branched-chain amino acids' OR 'amino acids, branched chain' OR 'branched-chain amino acid' OR 'acid,branched-chain amino' OR 'amino acid,branched-chain' OR 'branched chain amino acid' OR 'bcaa' OR 'isoleucine' OR 'leucine' OR 'valine''pre-diabetes':ab,ti

#3. #1 AND #2

#4. #3 AND 'human'/de

**Cochrane Library**

#1 MeSH descriptor: [Prediabetic State] explode all trees

#2 (State, Prediabetic):ti,ab,kw OR (States, Prediabetic):ti,ab,kw OR (Prediabetic States):ti,ab,kw OR (Prediabetes):ti,ab,kw OR (prediabetic):ti,ab,kw (Word variations have been searched)

#3 (insulin resistance):ti,ab,kw OR (HOMA-IR):ti,ab,kw OR (Impaired glucose tolerance):ti,ab,kw OR (glucose intolerance):ti,ab,kw OR (pre-diabetes):ti,ab,kw (Word variations have been searched)

#4 #1 or #2 or #3

#5 MeSH descriptor: [Amino Acids, Branched-Chain] explode all trees

#6 (Amino Acids, Branched-Chain):ti,ab,kw OR (Acids, Branched-Chain Amino):ti,ab,kw OR (Branched-Chain Amino Acids):ti,ab,kw OR (Amino Acids, Branched Chain):ti,ab,kw OR (Branched-Chain Amino Acid):ti,ab,kw (Word variations have been searched)

#7 (Acid, Branched-Chain Amino):ti,ab,kw OR (Amino Acid, Branched-Chain):ti,ab,kw OR (Branched Chain Amino Acid):ti,ab,kw OR (Amino Acids, Branched Chain):ti,ab,kw OR (Branched Chain Amino Acid):ti,ab,kw (Word variations have been searched)

#8 (BCAA):ti,ab,kw OR (Isoleucine):ti,ab,kw OR (leucine):ti,ab,kw OR (Valine):ti,ab,kw (Word variations have been searched)

#9 #5 or #6 or #7 or #8

#10 #4 and #9

**Web of Science**

1: TS= (prediabetic state OR insulin resistance OR HOMA-IR OR Impaired glucose tolerance OR glucose intolerance OR Prediabetic State* OR State, Prediabetic* OR States, Prediabetic OR prediabetic OR Prediabetes)

2: TS= (Amino Acid*, Branched-Chain OR Acid*, Branched-Chain Amino OR Branched-Chain Amino Acid* OR Amino Acid*, Branched Chain OR Branched-Chain Amino Acid OR Acid, Branched-Chain Amino OR mino Acid, Branched-Chain OR Branched Chain Amino Acid OR BCAA OR Isoleucine OR leucine OR Valine)

3: #1 AND #2

**REFERENCES**
